# Towards decentralized and sustainable water and wastewater treatment systems

**DOI:** 10.1038/s41598-025-93897-3

**Published:** 2025-04-25

**Authors:** Changsoo Lee, Lian-Shin Lin, Carlos A. Martínez-Huitle, Erica Pensini

**Affiliations:** 1 Department of Civil, Urban, Earth, and Environmental Engineering, Ulsan National Institute of Science and Technology (UNIST), Ulsan, 44919 Republic of Korea; 2 Wadsworth Department of Civil and Environmental Engineering, West Virginia University, PO BOX 6103, Morgantown, WV 26506-6103 USA; 3 Renewable Energies and Environmental Sustainability Research Group, Institute of Chemistry, Federal University of Rio Grande do Norte, Campus Universitário, Av. Salgado Filho 3000, Lagoa Nova, Natal, Rio Grande do Norte CEP 59078-970 Brazil; 4 School of Engineering, University of Guelph, Room 2525 Richards Bld., 50 Stone Road East, Guelph, ON N1G 2W1 Canada

**Keywords:** Environmental sciences, Pollution remediation

## Abstract

Achieving "sustainable management of water and sanitation for all" is a fundamental part of the Sustainable Development Goals (SDGs). Rising rates of urbanization and industrialization necessitate continual technological advancements to address existing and emerging water pollution issues related to centralized populations and industrial wastes. The articles in the Collection “Water and Wastewater Treatment Technologies” showcase salient aspects of advances in measurement and monitoring, as well as recommendations for sustainable use and management of water resources. The Collection associated with this editorial also presents studies that highlight the importance of decentralization and digitalization of environmental water technologies.

## Introduction

The present water crisis will probably worsen in the coming decades, stimulating research to identify and develop eco-friendly and efficient water and wastewater treatment solutions. Conventional water technologies, while delivering in some cases acceptable treatment performance, may use potentially hazardous chemicals, have high energy costs, or be operationally intensive. Recent developments in water and wastewater treatment technologies have shown promising advances in environmental remediation, monitoring, and capabilities for the abatement of persistent pollutants, as well as cost-competitiveness as compared to older technologies^1^. Recent research focuses on the development of water and wastewater technologies, emphasizing the fundamentals and new approaches, as well as the advances and challenges in translating these environment-friendly technologies from the laboratory to full-scale industrial/commercial devices^2–9^.

### Advances in decontamination

Significant public benefits might be gained by removing a wide range of pollutants and waterborne agents from various water matrices. Fundamental advances in decontamination efficiency and hybrid technologies suggest that these have the potential to help address the problem of water security. One such study performed by Wujcicki and co-workers^2^ presented an innovative method for removing phosphorus compounds from aqueous systems using chitosan-based hydrogel with dispersed cerium(IV) oxide (Ce-CTS). The modified chitosan enhanced physical and chemical adsorption, with a high phosphates(V) removal efficiency and rate. The adsorbent also demonstrated higher mechanical strength and better resistance to changeable chemical conditions than the CTS alone. This investigation will allow for rational design of alternative initial water pre-purification processes, resulting in more effective use of the adsorption capacity of the adsorbent.

In another decontamination study, Charazińska and colleagues^3^ investigated metal ion removal efficiency in industrial effluent using natural materials. While some materials, like orange peel and algae, were not effective enough, the potential use of a common plant, *Eclipta alba*, for removing ions including iron and chromium ions was established, with a removal rate of 95.3–97.6% and 97.4–99.9%, respectively.

Many water utilities aim to remove manganese from potable water supplies. Aesthetic concerns about water quality and possible distribution system issues, rather than public health concerns, are usually the driving forces behind manganese removal. A study performed by Earle and co-workers^4^ showed that raw surface water biofiltration can reduce effluent dissolved manganese, depending on dissolved oxygen concentrations and aeration for effective control. Then, this technology could replace conventional manganese treatments in regions without access to drinking water. However, larger media may be needed for adoption.

Co-contamination of industrial wastewater with organic solvents (*e.g.*, toluene and tetrahydrofuran) and metal ions (*e.g.*, Cu^2+^) is common in industrial wastewaters. The process developed by Earnden and colleagues^5^ focused on separating tetrahydrofuran (THF) from water and treating water polluted with either THF and copper or toluene and copper. In their method, lauric acid was used to facilitate the separation of THF from water, forming reverse micelles that lead to two distinct phases. On the one hand, the investigation demonstrated that lauric acid, when mixed directly with polluted water or solubilized in canola oil for injection into aquifers, can be effective in treating water contaminated with THF and Cu^2+^. On the other hand, injectable filters with cationic hydroxyethylcellulose downstream of pollutant plumes can impede the flow of toluene and copper ions, protecting downstream receptors.

### Advances in disinfection

Photocatalysis is the process of transforming the energy of photons into chemical energy with the use of a catalyst. A variety of materials, including metal oxides, polymers, clays, and doped metal oxides, can be used as photocatalysts in the treatment of industrial and domestic effluents. Taking a different approach, Hassanpour et al.^6^ presented a novel ultraviolet (UV) water disinfection process using concentric cylinders, with quartz inner cylinders and UV reflective outer cylinders. The results showed that this design avoids the fouling, and the maintenance challenges associated with traditional reactors, whilst maintaining the same or slightly better performance compared to traditional L-shaped UV photoreactors, making it a promising alternative.

Another UV-based study, performed by Song and colleagues^7^, focused on the use of 280 nm ultraviolet-light-emitting diodes (UV-LEDs) for disinfecting *M. abscessus* in water. The advantage of the method is that the approach is technically possible to deliver even with compact apparatuses for point-of-use (POU) applications. Inactivation kinetics modelling showed that *M. abscessus* is more resistant than *P. aeruginosa* and *L. pneumophila*, suggesting that nontuberculous mycobacteria are among the most UV-resistant opportunistic premise plumbing pathogens. The results confirm that an UV-LED disinfection unit design against these important pathogens in drinking water systems is a viable decentralized strategy to fulfill SDG 6 in different countries.

### Advances in detection

There is also a growing need for inexpensive devices that can rapidly and conveniently detect and monitor environmental pollutant levels on site to provide prompt and accurate information about the extent and quantity of pollutants. The ultimate objective is to develop inexpensive and portable laboratories that can be deployed in any setting. Over the past few years, there has been a significant surge in the utilization of smartphones for analytical purposes, because they provide a convenient wireless connection with other devices, particularly in the context of health and environmental applications. The study performed by Cardozo and co-workers^8^ described a decentralized imaging-based device for analyzing water pollution in terms of chemical oxygen demand (COD) and color. Their approach made use of the HSV (hue, saturation, value) and/or Red, Green and Blue (RGB) color model in a smartphone, and compared the data obtained with spectrophotometric measurements. The methods achieved an average accuracy of 96.2% for COD analysis with a spectrophotometer, and 98.3% for those captured by a smartphone camera. This smartphone-based analysis can be used for environmental examinations when spectrophotometers are unavailable by using technologies that are becoming much more commonplace. Investigations are underway to develop a smartphone application for environmental monitoring purposes and explore renewable energy sources.

Marine sediments contain higher levels of pollutants compared to the surrounding water, making them useful for identifying pollutants in the environment. Techniques that can quantitatively measure toxic substances in marine sediments with high sensitivity, accuracy, and cost-effectiveness are a promising focus for further research and new techniques are much required, since conventional methods have several drawbacks. Fernández et al.^9^ investigated the efficacy of an in-situ bismuth-modified carbon-fiber microelectrode as a voltamperometric sensor for simultaneous detection of lead (Pb), cadmium (Cd), and zinc (Zn) in marine sediments from Puerto Jeli in El Oro Province, Ecuador. The technique of differential pulse anodic stripping voltammetry with a range of measurements from 12 to 50 μg mL^−1^ was utilized to determine the concentration of metals. According to the preliminary results, Cd and Pb values exceeded the permissible limits established by both Ecuador and the US Environmental Protection Agency.

In summary, research tackling many questions in the field of water and wastewater treatment is continually contributing to advances towards SDG6—clean water and sanitation for all. We would like to take this opportunity to thank all authors for their original contributions, and particularly the reviewers, whose comments and suggestions were important to achieve high-quality article publications.
